# Custom-made fenestrated Anaconda™ leg extension internal iliac artery aneurysm: A case report

**DOI:** 10.1016/j.ijscr.2024.110790

**Published:** 2025-01-01

**Authors:** Georgios I. Karaolanis, Corinne Geppert, Konstantinos Kotopoulos, Drosos Kotelis, Vladimir Makaloski

**Affiliations:** a Department of Vascular Surgery, Inselspital, University Hospital, University of Bern, 3010 Bern, Switzerland; b Vascular Unit, Department of Surgery, University Hospital of Ioannina and School of Medicine, Ioannina, Greece

**Keywords:** Custom-fenestration, Iliac branch device alternatives, Endovascular treatment, Anaconda endograft, Aneurysm, Internal iliac artery

## Abstract

**Introduction and importance:**

Internal iliac artery aneurysms repair represents a life-threatening condition due to their anatomical position and the risk of rupture. Iliac branch devices are strongly recommended for anatomically suitable patients, but limited alternatives exist when their use is unsuitable. The use of custom-made fenestrated endografts is well documented in other aortic territories, however, their application for the treatment of internal iliac artery aneurysm remains limited.

**Case presentation:**

In this innovative technique case report, we demonstrate the use of a custom-made fenestrated Anaconda™ leg extension for treating an internal iliac artery aneurysm. In the present report, we describe this technique in a patient unfit for open repair and no suitable for iliac branch device (IBD) treatment.

**Clinical discussion:**

Iliac branch devices have been designed for the treatment of iliac artery aneurysms and are associated with high technical success rates and low patient morbidity. However, their use is unsuitable for certain anatomies, and alternative therapeutic options are sparsely reported in the literature. Custom-made devices represent a feasible treatment option for aortic aneurysm repair, but despite their success in other settings, their role in treating internal iliac artery aneurysms remains poorly defined.

**Conclusion:**

Custom made iliac fenestrated devices may be a valuable treatment option for patients not eligible for IBD devices.

## Highlights

• Custom made fenestrated endografts are widely accepted for the treatment of pararenal and thoracoabdominal aortic aneurysms
• Their application for preservation of the internal iliac artery are not widely reported
• Custom-made fenestrated Anaconda leg extension represents a feasible alternative for internal iliac artery aneurysm repair

## 1 Introduction

After three decades of innovation, endovascular aortic repair has become increasingly popular for the treatment of aortoiliac aneurysm repair [1]. Internal iliac artery aneurysms (IIAAs) are most commonly detected in association with other aortoiliac aneurysms, while only a small percentage present as isolated cases [1]. This life-threatening condition is clinically significant due to its non-specific symptoms, deep anatomic position and risk of rupture. Additionally, preservation of the IIA is well-documented to prevent unfavourable outcomes such as buttock claudication, colorectal ischemia and even spinal cord injury [1].

Multiple proposed solutions for IIA preservation include bell bottom techniques, stent graft placement and combined embolization with stent graft placement. Both the Society for Vascular Surgery and the European Society for Vascular Surgery, recommended the use of internal branch devices (IBD) in anatomically suitable patients [2,3]. Custom-made fenestrated endografts are widely used for treating pararenal and thoracoabdominal aortic aneurysms, with encouraging results [4,5]. However, their application in patients with iliac artery aneurysms has not been widely reported. The current study demonstrates the use of a custom-made fenestrated Anaconda™ leg extension for treating an internal iliac artery aneurysm.

## 2 Case presentation

**Fig. 1:**
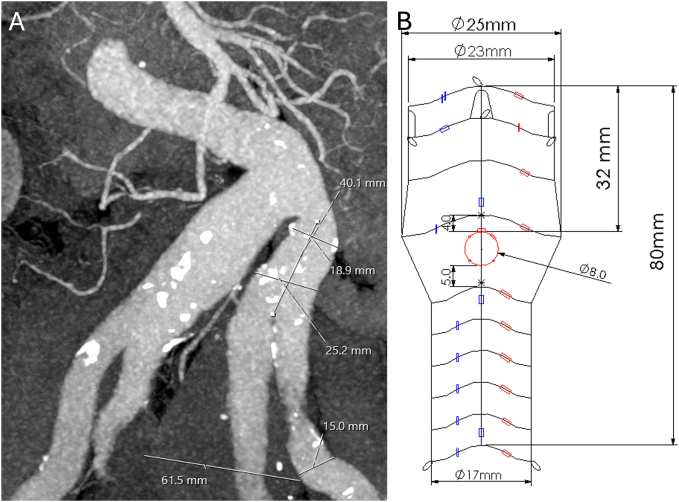
A) Preoperative CT-angiogram showing the anatomical features of the left common, internal and external iliac artery B) Technical drawing of the custom-made fenestrated leg extension.

A 76-year-old male was referred to our department with a left flank pain. A computed tomography angiography revealed 62 mm isolated left internal iliac artery aneurysm and significant kinking of the aorto-iliac segment. The anatomical measurements shown in Fig. 1A, ruled out the use of an off-the-shelf IBD. We planned an endovascular approach using a custom-made fenestrated Anaconda™ (Vascutek, Inchinnan, UK) leg extension (Fig. 1B) due to the lack of an adequate proximal sealing zone in the internal iliac artery. Additionally, our aim was to avoid the need for endovascular aortic aneurysm repair (EVAR) as the abdominal aorta diameter was within normal limits. Informed consent for publication of this case was obtained.

**Fig. 2:**
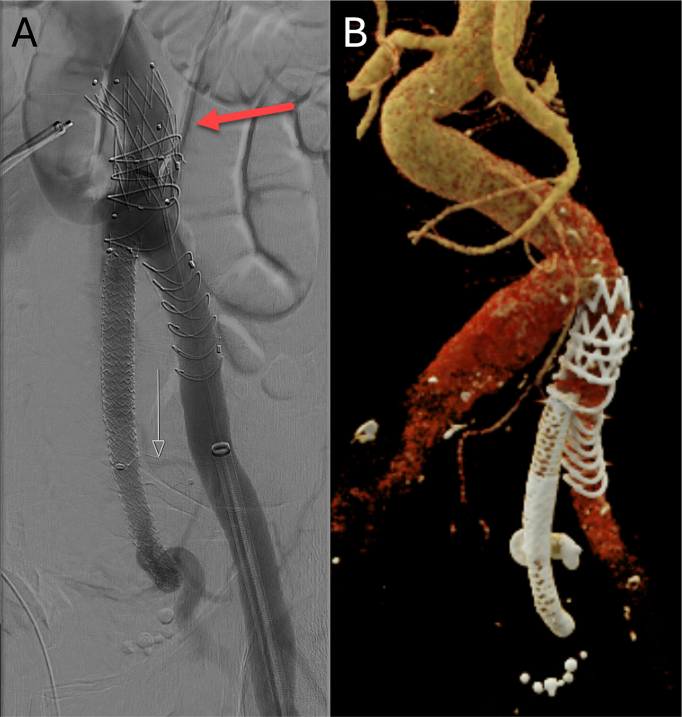
A) Final intraoperative angiography showing an excluded internal iliac artery aneurysm with proximal cuff (red arrow) and Amplatzer plug (white arrow) B) 3-D cinematic rendering. (For interpretation of the references to colour in this figure legend, the reader is referred to the web version of this article.)

Percutaneous bilateral femoral access was established. An angiogram from the left groin revealed the left-sided iliac anatomy. The custom-made fenestrated Anaconda™ leg extension was inserted and deployed in the left common iliac artery, with the fenestration facing on the take off of the internal iliac artery. From the contralateral groin, catheterization of the endograft's fenestration and the internal iliac artery was performed. To achieve a distal landing zone in the internal iliac artery, one internal artery side branch (superior gluteal artery) was occluded using a 14 mm Amplatzer plug (Abbott Laboratories, NY, USA) (Fig. 2A, white arrow) providing both proximal and distal sealing (inferior gluteal artery) with three Advanta V12 (Atrium Maquet, Getinge Group, Mijdrecht, the Netherlands) covered stents. (Fig. 2A) Intraoperative angiography revealed a low-flow type Ia endoleak, which was sealed using a 26x42mm proximal cuff (Cordis Medical GmbH). (Fig. 2A, red arrow). The completion angiogram confirmed complete isolation of the aneurysm and patency of the internal iliac artery without detection of any endoleak. Contrast volume used was 54 ml and total fluoroscopy time was 124 min. The patient was discharged at third postoperative day. A follow-up angio-CT scan at 28 months showed patent stent grafts with no evidence of endoleak. (Fig. 2B).

## 3 Clinical discussion

The ongoing advancement of the endovascular materials has increased the number of anatomies treatable via an endovascular approach. Iliac branch devices have been designed for the treatment of iliac artery aneurysms and are associated with high technical success rates and low patient morbidity, if the anatomy is suitable [2]. Moreover, the preservation of the internal iliac artery leads to a reduced incidence of adverse events, including buttock claudication, colonic ischaemia, pelvic necrosis or erectile dysfunction [2]. However, some patients present with anatomy unsuitable to these devices, and the rationale to embolize the internal iliac artery is obscured by potential complications [2,6,7]. Custom-made devices represent a feasible treatment option for pararenal and thoracoabdominal aortic aneurysms with high technical success rate [2]. Despite their success in other settings, the role of custom-made devices in treating internal iliac artery aneurysms remain poorly defined, particularly when alternative options are unavailable.

In the current study, the reliance on the presented technique using a custom-made leg extension device limits its feasibility in settings unsuitable for IBD. Beyond the message of the present study, it may be a worthwhile topic for further investigation to assess the ideal treatment of this group of patients with internal iliac artery aneurysms.

## 4 Conclusion

A Custom-made fenestrated leg extension can be safely performed, making it an excellent additional option for treating an internal iliac artery aneurysm when the anatomy is unsuitable for an off-the-shelf iliac branch device. Further high-volume prospective studies with longer follow-up periods are required to obtain robust data.

## CRediT authorship contribution statement

G.I.K: study concept and design, data collection, writing the paper

C.G: data collection and interpretation

K.K: data collection, writing the paper

D.K: study concept, supervise the paper

V.M: study concept, writing the paper and supervise the paper

## Consent

Written informed consent was obtained from the patient's family for publication of this case report and accompanying images. A copy of the written consent is available for review by the Editor-in-Chief of this journal on request.

## Ethical approval

The study is exempt from ethnical approval in our institution in Inselspital University Hospital of Bern, Switzerland due to the nature of the manuscript, which is case report.

## Guarantor

Georgios I. Karaolanis MD, MSc, PhD

## Funding

This research did not receive any specific grant from funding agencies in the public, commercial, or not-for-profit sectors.

## Methods section

The work has been reported in line with the SCARE criteria [8].

## Declaration of competing interest

The authors declare that there is no conflict of interest for the publication of this article.
